# The Spread of Infectious Diseases by Schools

**Published:** 1915-09

**Authors:** 


### THE SPREAD OF INFECTIOUS DISEASES BY SCHOOLS.

According to an editorial writer in *American Medicine*, the increasing opinion that infections of childhood are generally if not always contracted by direct contact with a prior case of carrier, has put a new weapon in the hands of those who object to schooling at early ages. The younger the child the more severe are all these diseases, whooping cough, for instance, being fatal to one in four attacked in the first year, and being almost harmless at twelve to fifteen. Indeed, at fifteen we are beginning to dismiss these diseases as a source of worry. The longer, then, we can keep the children out of school, the more chance they have to develop properly and reach maturity.

Nevertheless, we doubt whether this knowledge will have the slightest effect on the tendency to begin school earlier and earlier. Medical inspection will be depended upon to recognize and isolate the infected. As a matter of fact, in spite of occasional condemnation of schools as places for the swapping of infections, we are credibly informed that remarkably few cases are traced to schoolroom infection. They are infected from companions in other places, as they are well separated in the schoolroom. Sunday-schools are coming in for much of the blame, and though there is closer contact than in day schools, the churches exercise no precautions whatever, and have no medical inspectors.

The still closer contact in moving picture theaters has been accepted as the main reason for some recent epidemics, and health authorities in small communities are even closing the shows on the appearance of scarlet fever and diphtheria in town. This is not practicable in a large city where all the diseases of childhood are constantly present, but would it not be a good plan to exclude children up to ten or twelve anyhow, and up to sixteen if there is any special prevalence of disease in the neighborhood? Common sense would seem to dictate such a course.

